# Sex Differences in Cardiac Troponin I and T and the Prediction of Cardiovascular Events in the General Population

**DOI:** 10.1093/clinchem/hvab109

**Published:** 2021-07-08

**Authors:** Dorien M Kimenai, Anoop S V Shah, David A McAllister, Kuan Ken Lee, Athanasios Tsanas, Steven J R Meex, David J Porteous, Caroline Hayward, Archie Campbell, Naveed Sattar, Nicholas L Mills, Paul Welsh

**Affiliations:** 1 Usher Institute, University of Edinburgh, Edinburgh, UK; 2 BHF Centre for Cardiovascular Science, University of Edinburgh, UK; 3 Institute of Health and Wellbeing, University of Glasgow, Glasgow, UK; 4 Central Diagnostic Laboratory, Maastricht University Medical Center, Maastricht, the Netherlands; 5 CARIM School for Cardiovascular Diseases, Maastricht University, Maastricht, the Netherlands; 6 Institute of Genetics and Molecular Medicine, University of Edinburgh, Edinburgh, UK; 7 Institute of Cardiovascular & Medical Sciences, University of Glasgow, Glasgow, UK

**Keywords:** sex, cardiac troponin, risk factors, cardiovascular events

## Abstract

**Background:**

Cardiac troponin concentrations differ in women and men, but how this influences risk prediction and whether a sex-specific approach is required is unclear. We evaluated whether sex influences the predictive ability of cardiac troponin I and T for cardiovascular events in the general population.

**Methods:**

High-sensitivity cardiac troponin (hs-cTn) I and T were measured in the Generation Scotland Scottish Family Health Study of randomly selected volunteers drawn from the general population between 2006 and 2011. Cox-regression models evaluated associations between hs-cTnI and hs-cTnT and the primary outcome of cardiovascular death, myocardial infarction, or stroke.

**Results:**

In 19 501 (58% women, mean age 47 years) participants, the primary outcome occurred in 2.7% (306/11 375) of women and 5.1% (411/8126) of men during the median follow-up period of 7.9 (IQR, 7.1–9.2) years. Cardiac troponin I and T concentrations were lower in women than men (*P* < 0.001 for both), and both were more strongly associated with cardiovascular events in women than men. For example, at a hs-cTnI concentration of 10 ng/L, the hazard ratio relative to the limit of blank was 9.7 (95% CI 7.6–12.4) and 5.6 (95% CI 4.7–6.6) for women and men, respectively. The hazard ratio for hs-cTnT at a concentration of 10 ng/L relative to the limit of blank was 3.7 (95% CI 3.1–4.3) and 2.2 (95% CI 2.0–2.5) for women and men, respectively.

**Conclusions:**

Cardiac troponin concentrations differ in women and men and are stronger predictors of cardiovascular events in women. Sex-specific approaches are required to provide equivalent risk prediction.

## Introduction

Cardiovascular disease remains the main cause of death worldwide with 17.6 million people dying each year (1, 2). The development of approaches to improve the prediction and targeting of effective preventative therapies for those at highest risk may help minimize the impact of cardiovascular disease on the population. It is important that these approaches are equitable for women and men (2). In both primary and secondary care, guidelines have been established in populations where men are over represented and women seem to be disadvantaged and receive fewer preventative treatments (3–5).

Cardiac biomarkers may provide an unbiased aproach toward the prediction of cardiovascular events in women and men. Increasingly, high-sensitivity cardiac troponin is considered a useful marker of risk outwith the setting of acute coronary syndromes to evaluate asymptomatic individuals and guide therapeutic approaches to prevent the onset of cardiovascular disease (6–11). Recent major improvements in analytical performance have greatly enhanced assay sensitivity, such that with high-sensitivity assays we are now able to accurately measure cardiac troponin concentrations in the majority of healthy individuals (12). In a recent meta-analysis of apparently healthy individuals, 43% of participants with cardiac troponin concentrations in the top third developed cardiovascular disease over the next 8 years (13). 

It remains unclear in practice how best to harness this prognostic information to guide the use of primary and secondary prevention, and whether sex-specific troponin thresholds should be considered. The use of high-sensitivity assays has identified important differences in troponin concentrations between men and women, with the 99th centile upper reference limits used for diagnosis of myocardial infarction up to 2-fold higher in men (14). In our recent systematic review, we documented that this observation is consistent for all troponin assays across multiple cohorts from different ethnic backgrounds (15). Furthermore, we recently demonstrated in the Generation Scotland Scottish Family Health Study, where both cardiac troponin I and T were measured in the same cohort, that differences in the 99th centile between men and women exist for both biomarkers across all age groups (16). Although several studies have investigated cardiac troponins in relation to cardiovascular outcomes in the general population (7, 11, 13, 17, 18), few have evaluated how sex influences risk prediction, and it remains unclear whether a different approach is required in women and men. Our aim was to determine whether sex influences the predictive ability of cardiac troponin I and T for cardiovascular events in the general population.

## Material and Methods

### Study Population

The Generation Scotland Scottish Family Health Study is a well-phenotyped family-based cohort that enrolled 24 090 participants aged between 18 and 98 years and has been described previously (7, 16, 19). Briefly, individuals between 35 and 65 years of age were identified at random from participating general medical practices in Scotland between 2006 and 2011. Participants were asked to identify at least 1 first-degree relative who was at least 18 years of age that would also enrol. For this study, we excluded participants with missing cardiac troponin measurements. Study participants provided written informed consent, including linkage to their medical records. The study was conducted according to principles of the Declaration of Helsinki and was approved by the National Health Service Tayside Committee on Medical Research Ethics (REC Reference Number: 05/S1401/89). The study followed the Strengthening the Reporting of Observational Studies in Epidemiology (STROBE) reporting guideline.

### Clinical Characteristics

Participants completed a health questionnaire, and had physical characteristics and clinical characteristics measured according to a standardized protocol (19). Past medical history, including a diagnosis of diabetes mellitus, previous myocardial infarction or stroke, and use of medications was self-reported. Family history of cardiovascular disease was defined as a self-report of parents or siblings having heart disease or stroke. Blood samples were taken, according to a standard operating procedure, and serum was prepared. Total cholesterol, high-density lipoprotein cholesterol, and serum creatinine, were measured at the time of collection, and additional aliquots were stored at –80 °C for future analyses. The Scottish Index of Multiple Deprivation (2009) scores were derived from participants’ postcodes: they denote nationally compiled composite measures of small-area deprivation (20).

### Cardiac Troponin Measurements

Serum cardiac troponin I was measured on ARCHITECT *i*1000SR high-sensitivity cardiac troponin I assay (Abbott Diagnostics) and cardiac troponin T was measured on Cobas e411 high-sensitivity cardiac troponin T (Roche Diagnostics) assay. During the conduct of this study, we participated in the National External Quality Assurance Scheme (UKNEQAS) for these biomarkers. Both assays were calibrated and quality controlled using the manufacturer’s reagents. Coefficient of variations for cardiac troponin I were 6.2%, 6.0%, and 4.6% for the low, intermediate, and high control, respectively. Coefficients of variation for cardiac troponin T were 5.0% and 3.4% for the low and high control, respectively. Cardiac troponin T has a limit of blank (LoB) of 3 ng/L and limit of detection (LoD) of 5 ng/L. Cardiac troponin I has a LoB of 1.2 ng/L and LoD of 1.9 ng/L (21).

### Clinical Outcome

We used the Information Services Division National Health Service record linkage for Scotland to collect clinical outcome data until the end of September 2017. Information on cause of death was obtained using the National Health Service Central Register. Clinical outcomes were classified using the 10th revision of the International Classification of Diseases (ICD-10). The primary outcome was a composite endpoint of cardiovascular events including the following component endpoints: (*a*) cardiovascular death (I00 to I99), (*b*) myocardial infarction (I21, I22), and (*c*) stroke (I63, I64, G45). Secondary outcomes were cardiovascular death, noncardiovascular death, and all-cause death.

### Statistical Analysis

Continuous variables are presented as mean (SD) or median (25th–75th percentile), as appropriate. Categorical variables are presented as absolute numbers (%). For continous analyses, troponin values below the LoB were set to the LoB value divided by 2. The correlation between cardiac troponin I and T was assessed by Spearman correlation. Sex-specific incidence rates were calculated per 1000 person-years for clinical outcomes.

### Statistical Learning Using Cox Proportional Hazard Regression Models

Unadjusted and adjusted multiple fractional polynomial Cox proportional hazard regression analysis were conducted to quantify the relationship between cardiac troponin as a continuous variable with the primary outcome, stratified by sex. The multivariable model is adjusted for age, total cholesterol, high-density lipoprotein cholesterol, systolic blood pressure, cigarettes smoked per day, rheumatoid arthritis, diabetes mellitus, Scottish Index of Multiple Deprivation score, family history of cardiovascular disease, use of blood pressure medications, and use of cholesterol-lowering medications. Each continuous variable was chosen through backward-stepwise selection of the best fractional polynomial transformation. We created hazard ratio (HR) plots for the primary outcome at 5 years of the unadjusted and adjusted cardiac troponin I and T models for women and men, and evaluated the HR relative to the LoB. Due to the low proportion of missing covariates (<6%) and the large number of available samples we did not use imputation techniques, focusing on complete case analysis instead. We constructed receiver operating characteristic (ROC) curves and determined the area under the curve (AUC) to assess discrimination of cardiac troponin for predicting the primary outcome at 5 years in women and men. Individuals with no events by the 5-year mark were censored. Comparisons between unpaired AUCs were tested according to the DeLong method. In secondary analyses, we evaluated the HR relative to the LoD, and we evaluated the additional outcomes of cardiovascular death, noncardiovascular death, and all-cause death. All statistical analysis was performed using R v.3.6.2.

## Results

### Clinical Characteristics of Study Population

**Fig. 1.**
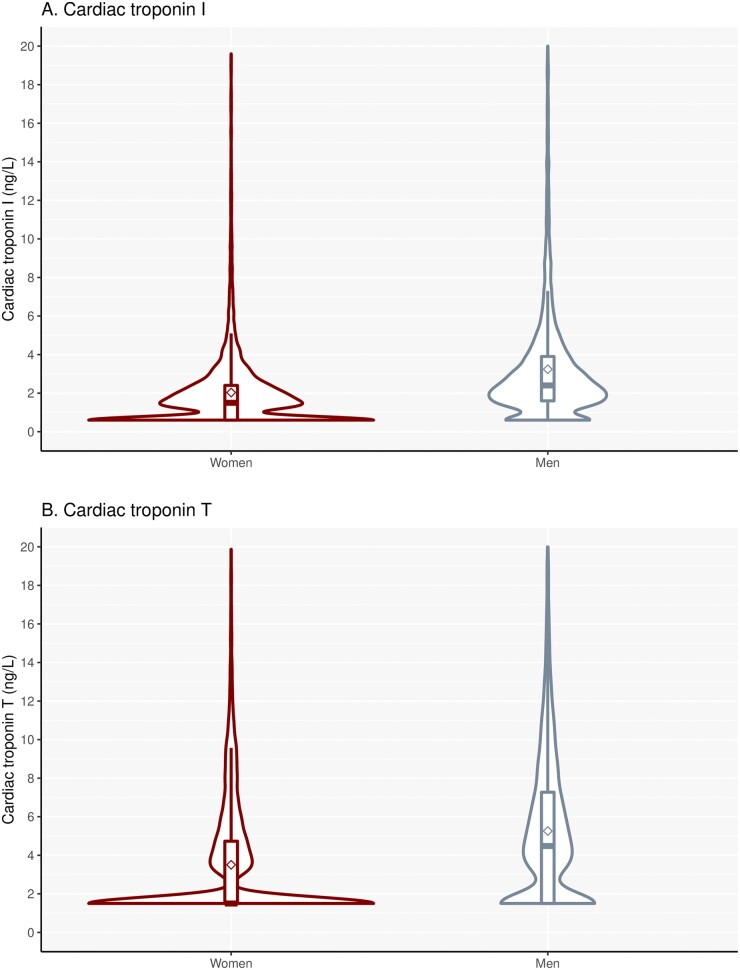
Distribution of cardiac troponins in women and men. Violin plots of cardiac troponin I (A) and T (B) distribution, stratified by sex (cardiac troponin I, women, 1.5 (1.2–2.5) ng/L versus men 2.5 (1.6–4.0) ng/L; cardiac troponin T, women ≤3.0 (3.0–4.8) ng/L versus men 4.6 (3.0–7.5) ng/L, *P* < 0.001 for both; *n* = 11 375 for women, and *n* = 8126 for men).

Our study population included 19 501 individuals (58% women; Table 1) with a measured cardiac troponin I and T concentration available. On enrolment women and men were at similar age (47 ± 15 years), but men were more likely to have risk factors, such as hypertension or diabetes mellitus, or to have a history of prior cardiovascular disease. Cardiac troponin concentrations were lower in women than men [cardiac troponin I, women 1.5 (1.2 to 2.5) ng/L versus men 2.5 (1.6 to 4.0) ng/L; cardiac troponin T, women ≤3.0 (3.0 to 4.8) ng/L versus men 4.6 (3.0 to 7.5) ng/L; *P* < 0.001 for both; Fig. 1]. The proportion of women and men with cardiac troponin I concentrations above the LoD was 66.1% (7523/11 375) and 86.8% (7056/8126), and for cardiac troponin T it was 42.4% (4826/11 375) and 68.5% (5569/8126). The correlation between cardiac troponin I and T concentrations was lower in women than men (*r* = 0.351, 95% CI 0.334 to 0.370 versus *r* = 0.446, 95% CI 0.428 to 0.463; *P* < 0.001).

**Table 1:** Baseline characteristics of entire study population and stratified by composite cardiovascular events.

	Study population		No incident cardiovascular event		Incident cardiovacular event	
	Women (*n* = 11 375)	Men (*n* = 8126)	Women (*n* = 11 069)	Men (*n* = 7715)	Women (*n* = 306)	Men (*n* = 411)
Age (years)	47 (15)	47 (15)	47 (15)	46 (15)	65 (14)	62 (11)
Body mass index (kg/m^2^)	26.5 (5.6)	26.9 (4.5)	26.5 (5.6)	26.8 (4.4)	27.8 (5.6)	28.3 (4.8)
Systolic blood pressure (mmHg)	128 (18)	136 (16)	128 (18)	136 (16)	141 (22)	142 (19)
Total cholesterol (mmol/L)	5.2 (1.1)	5.0 (1.1)	5.2 (1.1)	5.0 (1.06)	5.3 (1.3)	4.9 (1.2)
HDL cholesterol (mmol/L)	1.6 (0.4)	1.3 (0.3)	1.6 (0.4)	1.3 (0.3)	1.5 (0.4)	1.2 (0.4)
SIMD (score/10)	1.2 (0.7–2.4)	1.1 (0.7–2.1)	1.2 (0.7–2.4)	1.1 (0.7–2.1)	1.5 (0.8–2.9)	1.4 (0.8–2.6)
eGFR (mL/min/1.73 m^2^)	94 (17)	96 (17)	95 (17)	97 (17)	76 (20)	88 (25)
Cigarettes (per day)	2.3 (6.4)	2.7 (7.6)	2.2 (6.3)	2.6 (7.5)	4.3 (9.3)	4.9 (11.9)
Family history of CVD (yes)	4516 (40.4%)	2888 (36.5%)	4401 (40.5%)	2732 (36.4%)	115 (38.1%)	156 (38.5%)
Rheumatoid arthritis (yes)	213 (1.9%)	101 (1.2%)	195 (1.8%)	88 (1.1%)	18 (5.9%)	13 (3.2%)
Baseline CVD (yes)	369 (3.2%)	508 (6.3%)	302 (2.7%)	401 (5.2%)	67 (21.9%)	107 (26.0%)
Diabetes mellitus (yes)	256 (2.3%)	306 (3.8%)	228 (2.1%)	252 (3.3%)	28 (9.2%)	54 (13.1%)
Lipid-modifying medication (yes)	604 (5.3%)	678 (8.3%)	548 (5.0%)	587 (7.6%)	56 (18.3%)	91 (22.1%)
Antihypertensive medication (yes)	832 (7.3%)	742 (9.1%)	761 (6.9%)	646 (8.4%)	71 (23.2%)	96 (23.4%)
Cardiac troponin I (ng/L)	1.5 (1.2–2.5)	2.5 (1.6–4.0)	1.5 (1.2–2.4)	2.4 (1.6–3.9)	2.9 (1.8–5.9)	3.9 (2.3–7.3)
Cardiac troponin T (ng/L)	≤3.0 (3.0–4.8)	4.6 (3.0–7.5)	≤3.0 (3.0–4.7)	4.5 (3.0–7.3)	5.7 (3.0–10.5)	7.0 (3.8–12.2)
Detectable cardiac troponin I (≥1.2 ng/L)	7523 (66.1%)	7056 (86.8%)	7252 (65.5%)	6663 (86.4%)	271 (88.6%)	393 (95.6%)
Detectable cardiac troponin T (≥3.0 ng/L)	4826 (42.4%)	5569 (68.5%)	4610 (41.6%)	5238 (67.9%)	216 (70.6%)	331 (80.5%)

Categorical data are presented as n (%). Continuous variables are presented as mean (SD) or median (ranges, 25th–75th percentile), as appropriate. Abbreviatons: CVD, cardiovascular disease; eGFR, estimated glomerular filtration rate, HDL, high-density lipoprotein; SIMD, Scottish Index of Multiple Deprivation.

### Cardiac Troponins and Cardiovascular Events in Women and Men

The median follow-up period was 7.9 (7.1 to 9.2) years, and a total number of 717 (3.7%) individuals experienced a primary outcome event. In those participants with an incident cardiovascular event (Table 1), women were on average 3 years older than men (65 versus 62 years), but otherwise prior cardiovascular disease and risk factors were similar. Women had fewer events than men, with the primary outcome occurring in 306 (2.7%) women and 411 (5.1%) men during the follow-up period (Table 2).

**Table 2:** Incidence rates of clinical outcomes in women and men.

	Women (*n* = 11 375)		Men (*n* = 8126)	
	Total events (%)	Incidence rate	Total events (%)	Incidence rate
Composite cardiovascular event	306 (2.7%)	3.3/1000 person-years	411 (5.1%)	6.3/1000 person-years
Myocardial infarction	81 (0.7%)	0.9/1000 person-years	178 (2.2%)	2.7/1000 person-years
Ischemic stroke	93 (0.8%)	1.6/1000 person-years	112 (1.4%)	2.3/1000 person-years
Cardiovascular death	128 (1.1%)	1.4/1000 person-years	138 (1.7%)	2.1/1000 person-years
Noncardiovascular death	206 (1.8%)	2.2/1000 person-years	168 (2.1%)	2.5/1000 person-years
All-cause death	334 (2.9%)	3.6/1000 person-years	306 (3.8%)	4.6/1000 person-years

Composite cardiovascular event = myocardial infarction, ischemic stroke, or cardiovascular death.

**Fig. 2.**
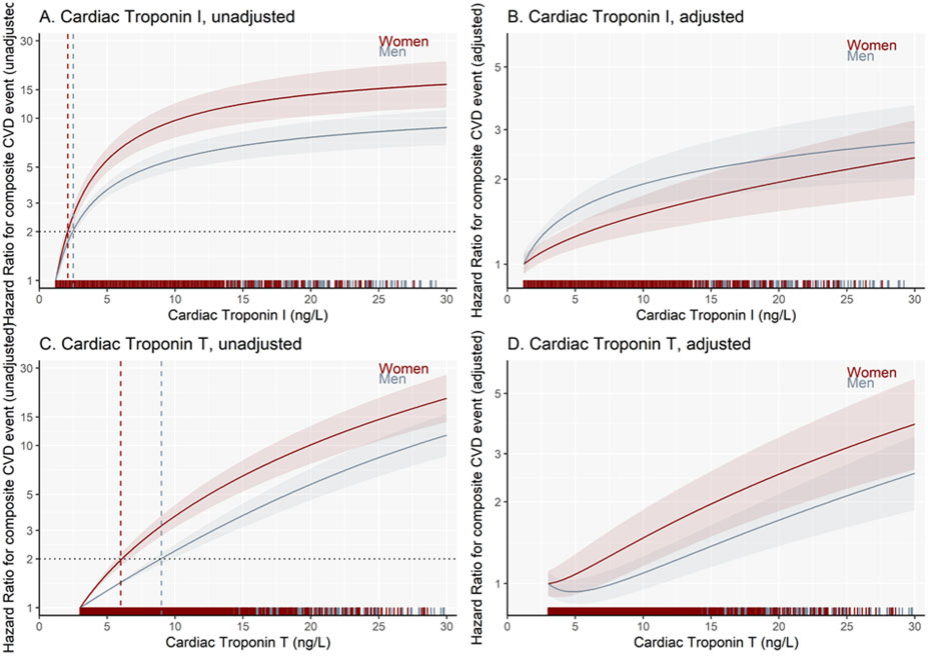
Hazard ratio plots for 5-year risk composite cardiovascular events. Troponin I: (A), unadjusted model; (B), adjusted model and T: (C), unadjusted model; and (D), adjusted model concentrations in relation to composite cardiovascular events, stratified by sex (referent = LoB value). The horizontal dashed line represents the doubling in risk of having a cardiovascular event within 5 years and the vertical dashed lines (red, women; gray, men) respresents the sex-specific thresholds of the 2-fold higher likelihood experiencing a cardiovascular event, accordingly.

Based on our unadjusted and adjusted regression models we illustrate the HR of a cardiovascular event at 5 years according to cardiac troponin I and troponin T concentrations in men and women (Fig. 2). For estimation of HRs, covariates were standardized for both women and men to illustrate the relationship between cardiac troponin and events in women and men with similar characteristics. Both cardiac troponin I and T concentrations were more strongly associated with the primary outcome in women than men (Fig. 2, A and C). For example, at a cardiac troponin I threshold of 10 ng/L, the unadjusted HR relative to the LoB was 9.7 (95% CI 7.6 to 12.4) for women compared to 5.6 (95% CI 4.7 to 6.6) for men. The unadjusted HR for a cardiac troponin T threshold of 10 ng/L relative to LoB was 3.7 (95% CI 3.1 to 4.3) for women and 2.2 (95% CI 2.0 to 2.5) for men. Cardiac troponin I and T thresholds of 2.1 ng/L and 6.0 ng/L, respectively, were associated with a doubling of cardiovascular risk in women. For men, a doubling of cardiovascular risk required higher thresholds of 2.5 ng/L and 9.0 ng/L for cardiac troponin I and T, respectively. Both cardiac troponin I and T remained strongly associated with cardiovascular events in women and men after adjustment of other risk factors, but the divergence between women and men was attenuated (Fig. 2, B and D).

**Fig. 3.**
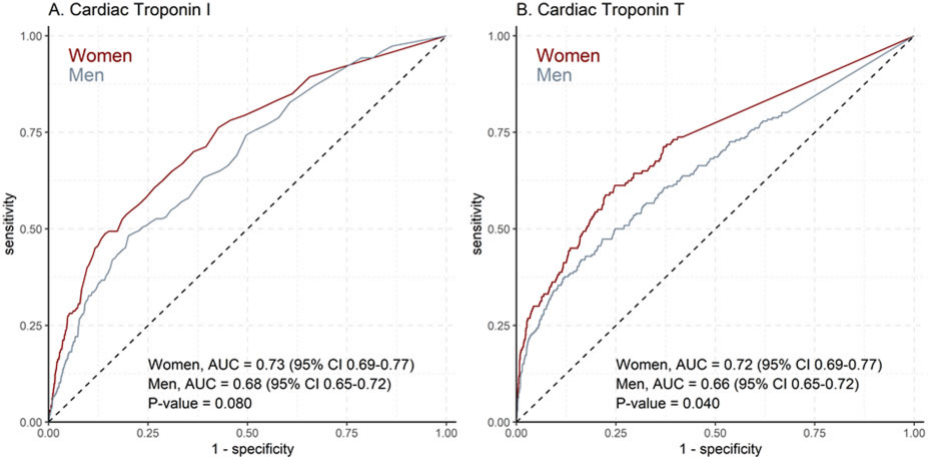
Comparison of the discrimination of cardiac troponins for the prediction of the composite cardiovascular event in women and men. Receiver-operating-curve for cardiac troponin I (A) and cardiac troponin T (B) to predict the composite cardiovascular event at 5 years in women and men.

Overall, cardiac troponin I and T concentrations had a good discriminative ability to predict 5-year cardiovascular risk (Fig. 3). For cardiac troponin I (AUC 0.73 in women versus AUC 0.68 in men, *P* = 0.080), and for cardiac troponin T (AUC 0.72 in women versus AUC 0.66 in men, *P* = 0.040) there was a trend toward better discrimination in women than in men.

### Secondary Outcomes

When using the LoD as reference value, we observed that the differences in the association with cardiac troponin I and T on the primary outcome between women and men were similar but attenuated (Fig. 1 in the online Data Supplement). Consistent with our observations for the primary composite outcome, the incidence of cardiovascular death was lower in women than men (Table 2). In contrast, no difference was observed in the incidence of noncardiovascular death between sexes. Both cardiac troponin I and T were strongly associated with cardiovascular death (online Supplemental Figs. 2 and 3, *P* < 0.001 for both) and all-cause death (online Supplemental Figs. 4 and 5, *P* < 0.001 for both) in women and men in fully adjusted models. Cardiac troponin I was not associated with noncardiovascular death in either women (*P* = 0.597) or men (*P* = 0.364), whereas cardiac troponin T was for both sexes (*P* = <0.001 in women, *P* = 0.004 in men; online Supplemental Figs. 6 and 7).

## Discussion

We have evaluated whether sex influences the prediction of cardiac troponin I and T for cardiovascular events in the general population. Our study has 3 main findings. First, cardiac troponin I and T are independent predictors of cardiovascular events in both women and men in the general population. Second, cardiac troponin concentrations differ between women and men and are stronger predictors of cardiovascular events in women. Use of the same thresholds to guide risk of future cardiovascular events in women and men would not provide equivalent prediction. Third, differences in prediction between women and men are largely explained by the prevalence of cardiovascular risk factors and prior disease, as the divergence between women and men was attenuated after adjustment of other risk factors. These findings highlight the importance of a sex-specific approach when using high-sensitivity cardiac troponin testing in isolation for risk stratification and targeting treatments to prevent cardiovascular disease. Ideally, cardiac troponin would be used as a continuous measure in a cardiovascular risk prediction tool that incorporates sex and other clinical features.

Our study has several strengths. First, the Generation Scotland Scottish Family Health Study enrolled approximately 20 000 individuals and a high proportion were women. Second, we were able to evaluate both cardiac troponin I and T in almost the entire cohort, permitting direct comparisons between markers and ensuring our findings are both representative and generalizable. Third, complete follow-up for almost 8 years ensured we had a sufficient number of cardiovascular events to evaluate prediction in men and women separately.

We found that cardiac troponins are strong independent predictors of cardiovascular events and that in their unadjusted, “raw” status, they are more strongly associated in women than men. This observation is in line with the Atherosclerosis Risk in Communities (ARIC) study, the Nord-Trøndelag Health Study (HUNT) study and the Activity and Function in the Elderly in Ulm (ActiFE) study, showing an interaction between troponin and sex in relation to future cardiovascular events across different ethnicities and age groups (18, 22, 23). Apart from differences in left ventricular mass that could explain the lower troponin concentrations in women than men (24–26), sex hormones may play a role in the divergent cardiovascular risk prediction of cardiac troponin concentrations for women and men (27). Estrogens seems to have a cardioprotective effect in premenopausal women, either directly or indirectly (28–31). Differences in body fat distribution between women and men may lead to a different cardiometabolic risk profile (32), which could influence cardiac troponin concentrations. Also, differences in the prevalence of microvascular disease may play a role (33, 34). However, in line with the ARIC study (22), ActiFE study (23), the Prospective Investigation of the Vasculature in Uppsala Seniors (PIVUS) study (35), the Age, Gene/Environment Susceptibility-Reykjavik (AGES-Reykjavik) study (36), and the Multi-Ethnic study of Atherosclerosis (MESA) (37), we showed that after adjustment of other risk factors the associations between cardiac troponins and outcome in women and men became similar. This points out that the divergent risk between sexes is at least partly explained by differences in cardiovascular risk profile. We determined previously that age, diabetes, prior cardiovascular disease, and lipid-lowering and antihypertensive medication use are important determinants for elevated cardiac troponin concentrations (16), and adjusting for these factors resulted in similar risk prediction for cardiac troponins in women and men.

What are the implications of these observed sex differences? Women tend to be undertreated for cardiovascular risk (38), and cardiac troponin might be a tool to bridge this imbalance. We believe that differences in prediction between women and men are largely explained by the prevalence of cardiovascular risk factors and prior disease, and therefore ideally cardiac troponin would be used as a continuous measure in a risk prediction tool that incorporates sex and other clinical features. However, if cardiac troponin is used in isolation to stratify patients into low- or high-risk groups for screening purposes, it is essential that a sex-specific approach is adopted. We believe that future research should focus on using cardiac troponin as a continuous variable in a multivariable cardiovascular risk tool that stratifies individuals based on their likelihood of cardiovascular disease. This would be in line with the development of the use of troponins in the acute cardiac setting (8), as the awareness has been raised that consideration of cardiac troponin in a continuous fashion improves risk assessment. Another major advantage of such an approach is that cardiac troponin could be corrected for other relevant risk factors and that would eliminate the problem of under- or overestimation for other important subgroups apart from sex.

In contrast to the acute care setting, the general population contains a high proportion of individuals with cardiac troponin values below the LoD. As our study was not designed to develop a risk prediction tool, but rather to evaluate sex differences between troponins in this setting, we have used cardiac troponins over their entire concentration range. We therefore cannot exclude that the imprecision profile of these assays in individuals with very low cardiac troponin concentrations may have affected the accuracy of our results. When using the LoD rather than the LoB as the reference, differences between women and men were less pronounced. Women have lower troponin concentrations than men and therefore a greater proportion of women have undetectable cardiac troponin concentrations. Our analyses suggest that discrimination in the modeling of future cardiovascular events is partly dependent on being able to identify those individuals who are very low risk with the lowest cardiac troponin values. The clinical implications of this are important. For example, in the USA, cardiac troponin values below the LoD are not reported because of concerns about assay imprecision. While precision is greater in those with higher values and therefore the user can be more confident in actioning the results of those identified as higher risk, it is less clear that based on current analytical precision (and reporting requirements) we are fully harnessing the potential of these tests to identify those who are lower risk. Those developing clinical tools to guide primary prevention approaches that incorporate cardiac troponin should be aware that including troponin values below the LoD may affect the accuracy of prediction and limit the future application of these tools in practice.

Another important observation in our study is that cardiac troponin T, but not cardiac troponin I predicts noncardiovascular death in both women and men. This extends our previous finding that cardiac troponin I has a greater specificity for future cardiovascular risk (7, 16). Although the underlying mechanism of this divergence is not well understood and remains speculative, cardiac troponin T elevations appear more strongly related to chronic kidney and neuromuscular diseases (39, 40). Furthermore, the curvilinear relationships between cardiovascular risk and cardiac troponin I and T concentrations differ. For cardiac troponin I, the risk increases in the low troponin range, while for cardiac troponin T the risk accelerates more at higher cardiac troponin values. This divergence may reflect differences in assay precision at very low concentrations and could be an important consideration for the development and implementation of risk prediction tools incorporating troponin, as model performance is likely to be very sensitive to assay choice.

Several limitations merit attention. First, no cardiac imaging data were available and studying the possible structural microvascular cardiac differences between women and men in relation to troponin and outcome was not possible, although this is of secondary value as we have incident cardiovascular outcomes. Second, most of the Generation Scotland Family Health Study participants are Caucasian and generalizing our findings to other ethnic groups should be done with caution. Third, although high-sensitivity testing was used, still a high proportion of individuals had undetectable cardiac troponin concentrations, particularly for cardiac troponin T and particularly in women. Imprecision in those with very low cardiac troponin concentrations might have influenced the accuracy of our model estimates. Finally, cardiac troponin I was only measured using one manufacturer’s assay, which precludes the direct extrapolation of our findings to other cardiac troponin I assays.

In conclusion, cardiac troponin I and T are independent predictors of cardiovascular events in both women and men in the general population. Cardiac troponin concentrations differ in women and men and are stronger predictors of cardiovascular events in women. Sex-specific approaches are required to provide equivalent risk prediction when using high-sensitivity cardiac troponin testing in isolation for risk prediction and the prevention of cardiovascular disease. Ideally, cardiac troponin would be used as a continuous measure in a cardiovascular risk prediction tool that incorporates sex and other clinical features.

## Supplemental Material

Supplemental material is available at *Clinical Chemistry* online

## Supplementary Material

hvab109_Supplementary_Data

## Employment or Leadership

None declared.

## Consultant or Advisory Role

N. Sattar, Amgen, AstraZeneca, Boehringer Ingelheim, Eli Lilly, Merck Sharp & Dohme, Novartis, Novo Nordisk, Pfizer, and Sanofi; N.L. Mills, Abbott Diagnostics, Roche, Beckman-Coulter, and Singulex.

## Stock Ownership

None declared.

## Honoraria

A.S.V. Shah, Abbott Diagnostics. S.J.R. Meex, lecture fees from Abbott Diagnostics and Roche Diagnostics; N. Sattar, Amgen, AstraZeneca, Boehringer Ingelheim, Eli Lilly, Merck Sharp & Dohme, Novartis, Novo Nordisk, Pfizer, Sanofi.

## Expert Testimony

None declared.

## Patents

None declared.

## Other Remuneration

N. Sattar, support for attending meetings and/or travel from Amgen, AstraZeneca, Boehringer Ingelheim, Eli Lilly, Novo Nordisk, Pfizer, Sanofi.

## Nonstandard Abbreviations:

Hs-cTn: high-sensitivity cardiac troponin
STROBE: Strengthening the Reporting of Observational Studies in Epidemiology
LoB: limit of blank
LoD: limit of detection
ICD-10: 10th revision of the International Classification of Diseases
HR: hazard ratio
ROC: receiver operating characteristic
AUC: area under the curve
CI: confidence interval
ARIC: Atherosclerosis Risk in Communities study
HUNT: Nord-Trøndelag Health Study
ActiFE: Activity and Function in the Elderly in Ulm study
PIVUS: Prospective Investigation of the Vasculature in Uppsala Seniors study
AGES-Reykjavik: Age, Gene/Environment Susceptibility-Reykjavik study
MESA: Multi-Ethnic study of Atherosclerosis study
