# Supplementary material for: Sex Differences in Cardiac Troponin I and T and the Prediction of Cardiovascular Events in the General Population
Source: Clin Chem. 2021 Jul 8;67(10):1351–60. doi: 10.1093/clinchem/hvab109 (PMC8486023; doi:10.1093/clinchem/hvab109)
Supplement: hvab109_Supplementary_Data [file hvab109_supplementary_data.docx]

SUPPLEMENTAL MATERIAL

**Sex Differences in Cardiac Troponin I and T and the Prediction of Cardiovascular Events in the General Population**

Dorien M Kimenai^a^, Anoop SV Shah^a,b^, David A McAllister^c^, Kuan K Lee^b^, Athanasios Tsanas^a^, Steven JR Meex^d,e^, David Porteous^f^, Caroline Hayward^f^, Archie Campbell^f^, Naveed Sattar^g^, Nicholas L Mills^a,b^, Paul Welsh^g^

**Running Title:** Sex Differences in Cardiac Troponin I and T

^a^Usher Institute, University of Edinburgh, Edinburgh, United Kingdom;

^b^ BHF Centre for Cardiovascular Science, University of Edinburgh, United Kingdom;

^c^ Institute of Health and Wellbeing, University of Glasgow, Glasgow, United Kingdom;

^d^ Central Diagnostic Laboratory, Maastricht University Medical Center, Maastricht, The Netherlands;

^e^ CARIM School for Cardiovascular Diseases, Maastricht University, Maastricht, the Netherlands;

^f^ Institute of Genetics and Molecular Medicine, University of Edinburgh, Edinburgh, United Kingdom;

^g^ Institute of Cardiovascular & Medical Sciences, University of Glasgow, Glasgow, United Kingdom

**Corresponding author:**

Professor Nicholas L Mills

BHF/University Centre for Cardiovascular Science

The University of Edinburgh

Edinburgh EH16 4SA

United Kingdom

Telephone: +44 131 242 6515

Fax: +44 131 242 6379

Email: [nick.mills@ed.ac.uk](mailto:nick.mills@ed.ac.uk)


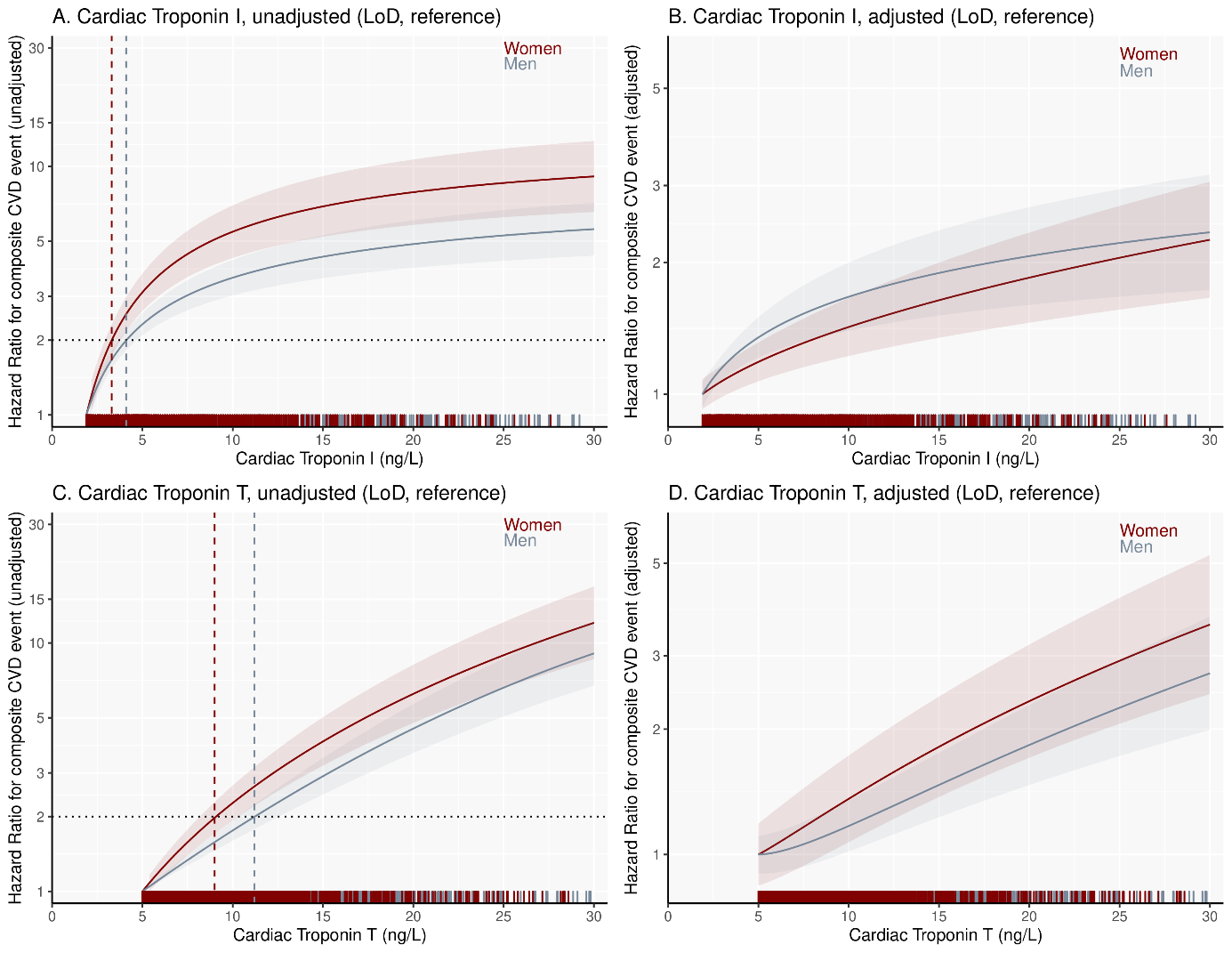


**Supplemental Figure 1. Hazard ratio plots for 5-year risk composite cardiovascular events.** Troponin I (A: unadjusted model; B: adjusted model) and T (C: unadjusted model; D: adjusted model) levels in relation to composite cardiovascular events, stratified by sex (referent = LoD value). The horizontal dashed line represents the doubling in risk of having a cardiovascular event within 5 years and the vertical dashed lines (red: women; grey: men) respresents the sex-specific thresholds of the two-fold higher likelihood experiencing a cardiovascular event, accordingly.

**
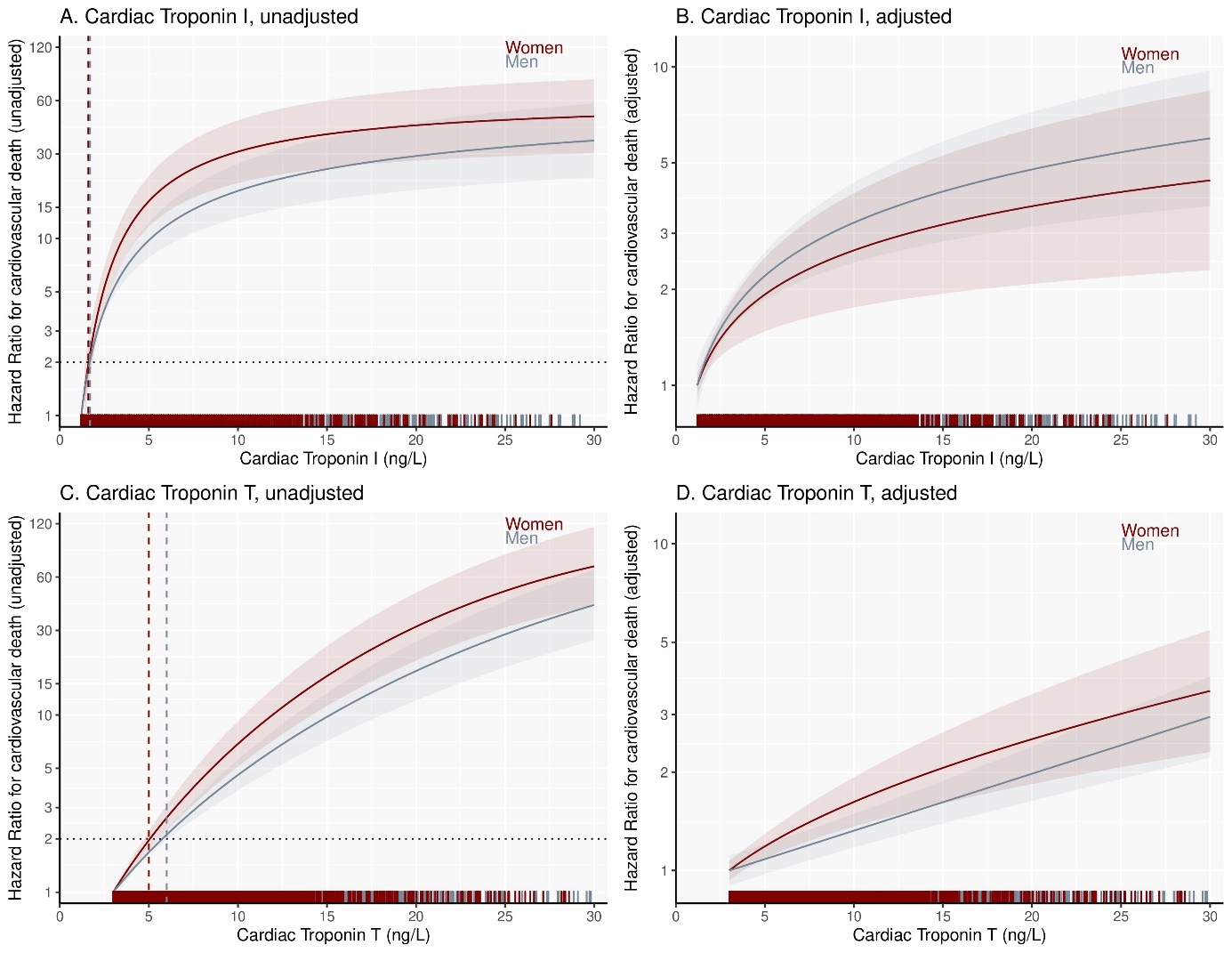
**

**Supplemental Figure 2. Hazard Ratio plots for 5-year risk cardiovascular death.** Cardiac troponin I (Panel A: unadjusted model; Panel B: adjusted model) and cardiac troponin T (Panel C: unadjusted model; Panel D: adjusted model) levels in relation to cardiovascular death, stratified by sex. HR ratio plot represents the risk of cardiovascular death within 5 years per ng/L increase in cardiac troponin levels in the average study population (referent = limit of blank value). The horizontal dashed line represents the doubling in risk of cardiovascular death within 5 years and the vertical dashes lines (red: women; grey: men) represents the sex-specific thresholds of the two-fold higher likelihood of cardiovascular death, accordingly. Adjusted models are adjusted for age, total cholesterol, high-density lipoprotein cholesterol, systolic blood pressure, cigarettes smoked per day, rheumatoid arthritis, diabetes mellitus, Scottish Index of Multiple Deprivation score, family history of cardiovascular disease, baseline cardiovascular disease, use of blood pressure medications, and use of cholesterol-lowering medications.

**
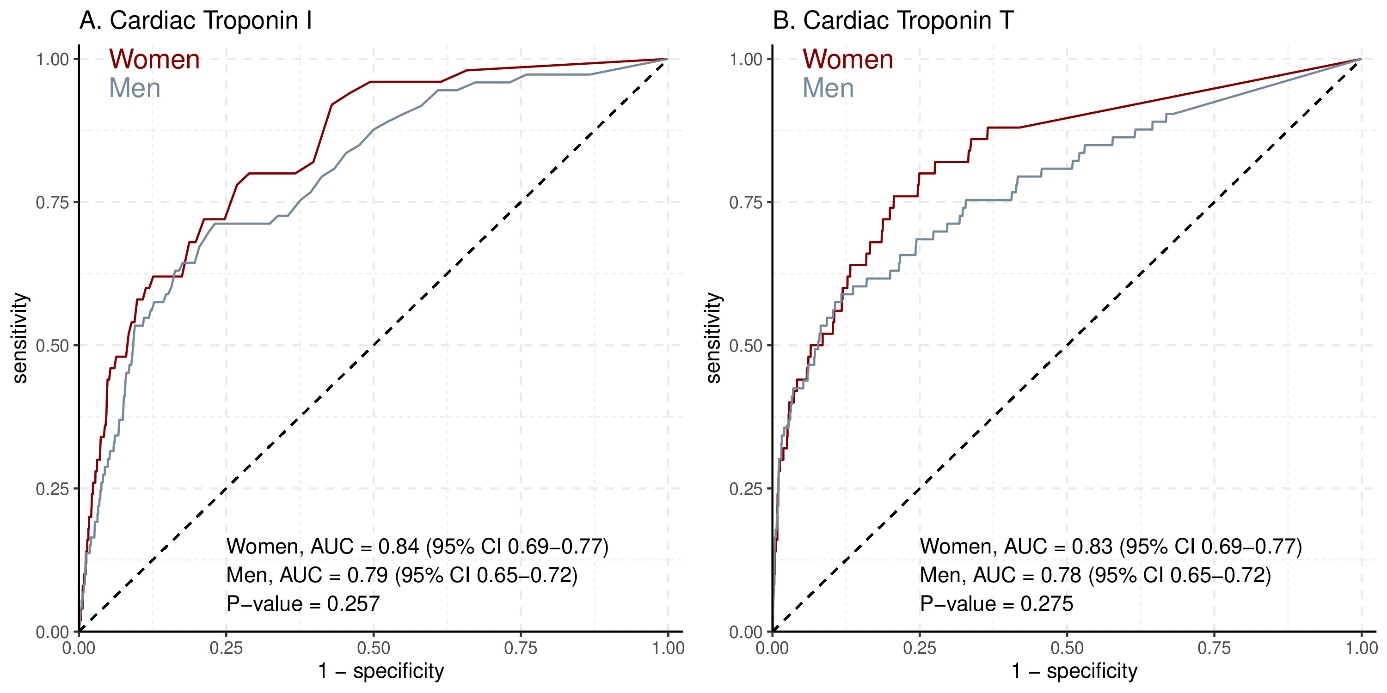
**

**Supplemental Figure 3. Comparison of the discrimination of cardiac troponins for the prediction of cardiovascular death in women and men.** Receiver-operating-curve for cardiac troponin I (Panel A) and cardiac troponin T (panel B) to predict cardiovascular death at 5 year in women and men.

**
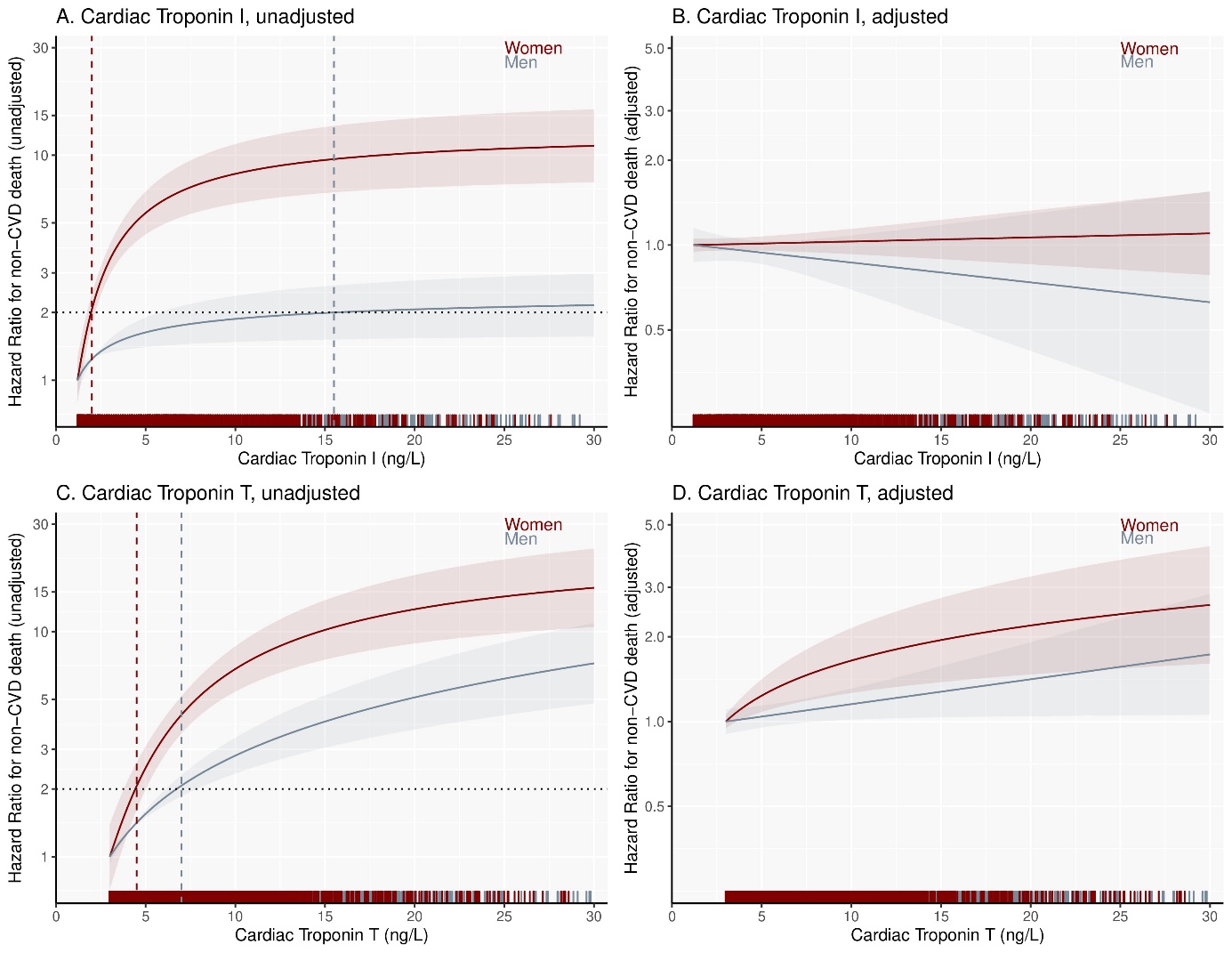
**

**Supplemental Figure 4. Hazard Ratio plots for 5-year risk non-cardiovascular death.** Cardiac troponin I (Panel A: unadjusted model; Panel B: adjusted model) and cardiac troponin T (Panel C: unadjusted model; Panel D: adjusted model) levels in relation to non-cardiovascular death, stratified by sex. HR ratio plot represents the risk of non-cardiovascular death within 5 years per ng/L increase in cardiac troponin levels in the average study population (referent = limit of blank value). The horizontal dashed line represents the doubling in risk of non-cardiovascular death within 5 years and the vertical dashes lines (red: women; grey: men) represents the sex-specific thresholds of the two-fold higher likelihood of non-cardiovascular death, accordingly. Adjusted models are adjusted for age, total cholesterol, high-density lipoprotein cholesterol, systolic blood pressure, cigarettes smoked per day, rheumatoid arthritis, diabetes mellitus, Scottish Index of Multiple Deprivation score, family history of cardiovascular disease, baseline cardiovascular disease, use of blood pressure medications, and use of cholesterol-lowering medications.

**
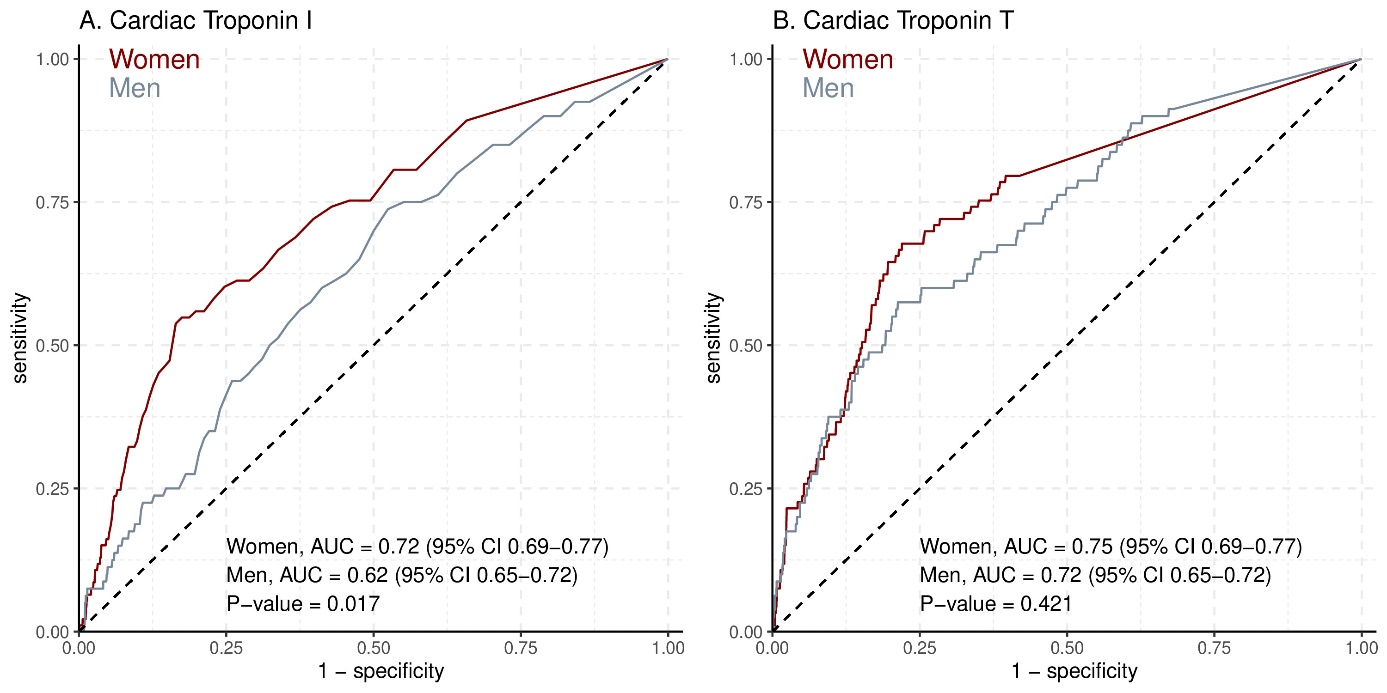
**

**Supplemental Figure 5. Comparison of the discrimination of cardiac troponins for the prediction of non-cardiovascular death in women and men.** Receiver-operating-curve for cardiac troponin I (Panel A) and cardiac troponin T (panel B) to predict non-cardiovascular death at 5 year in women and men.

**
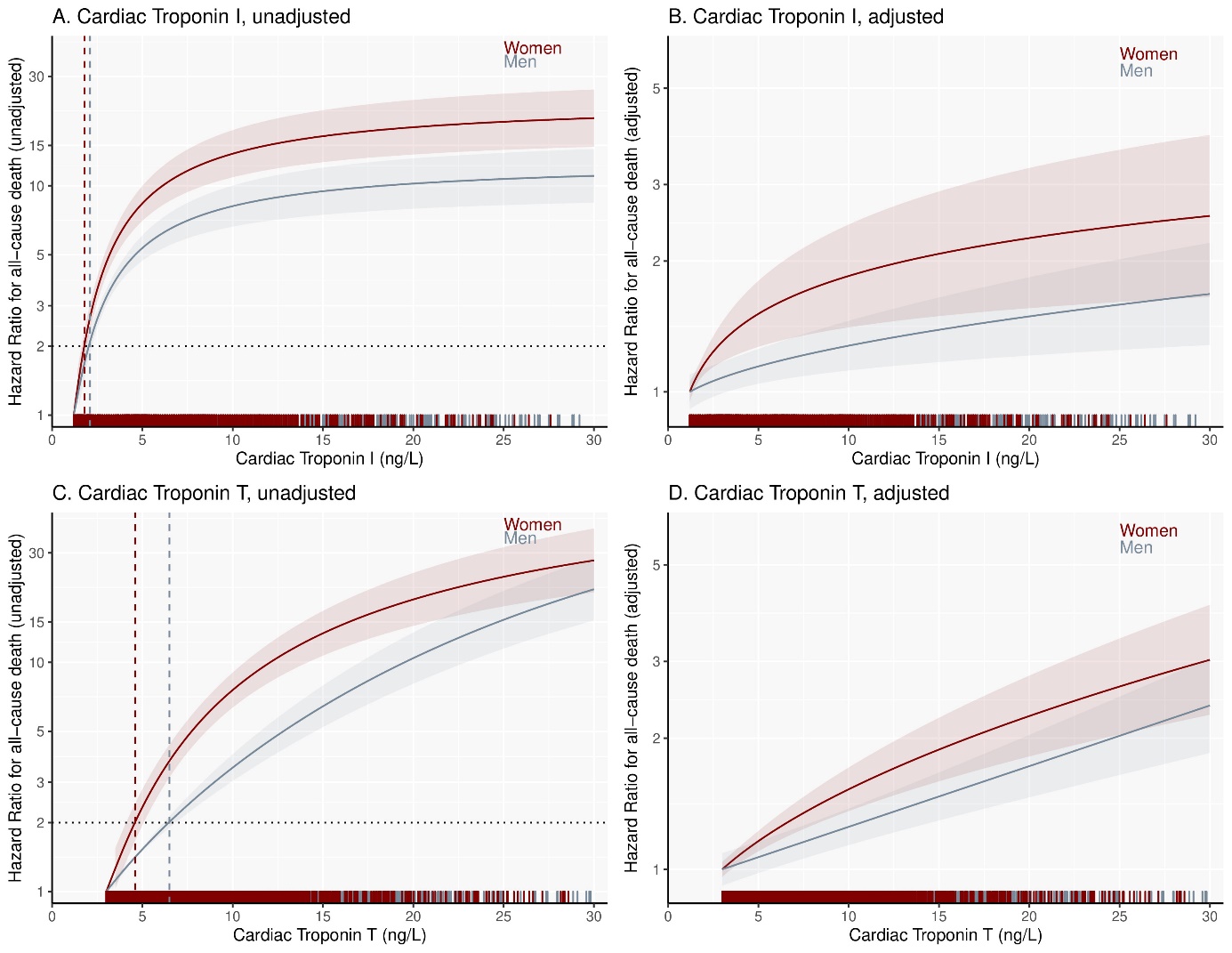
**

**Supplemental Figure 6. Hazard Ratio plots for 5-year risk all-cause death.** Cardiac troponin I (Panel A: unadjusted model; Panel B: adjusted model) and cardiac troponin T (Panel C: unadjusted model; Panel D: adjusted model) levels in relation to all-cause death, stratified by sex. HR ratio plot represents the risk of all-cause death within 5 years per ng/L increase in cardiac troponin levels in the average study population (referent = limit of blank value). The horizontal dashed line represents the doubling in risk of all-cause death within 5 years and the vertical dashes lines (red: women; grey: men) represents the sex-specific thresholds of the two-fold higher likelihood of all-cause death, accordingly. Adjusted models are adjusted for age, total cholesterol, high-density lipoprotein cholesterol, systolic blood pressure, cigarettes smoked per day, rheumatoid arthritis, diabetes mellitus, Scottish Index of Multiple Deprivation score, family history of cardiovascular disease, baseline cardiovascular disease, use of blood pressure medications, and use of cholesterol-lowering medications.

**
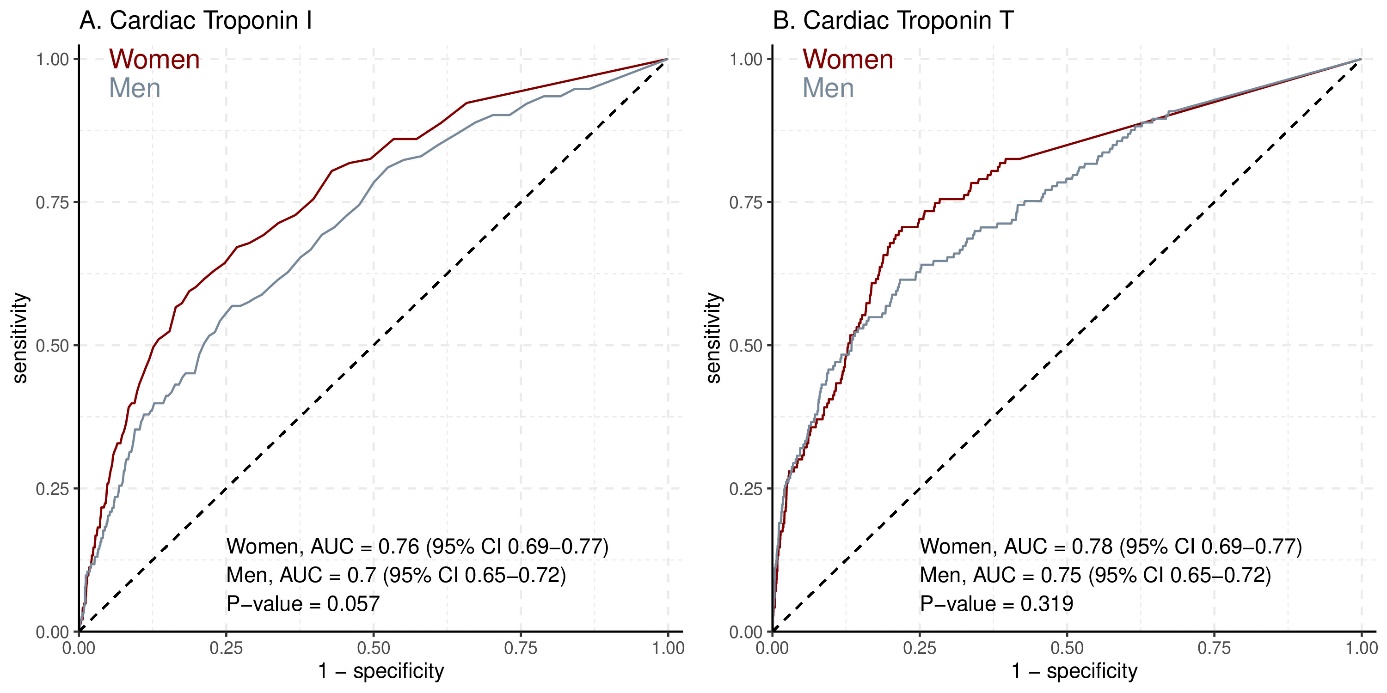
**

**Supplemental Figure 7. Comparison of the discrimination of cardiac troponins for the prediction of all-cause death in women and men.** Receiver-operating-curve for cardiac troponin I (Panel A) and cardiac troponin T (panel B) to predict all-cause death at 5 year in women and men.
